# Retraction: LncRNA FBXL19-AS1 promotes breast cancer cells proliferation and invasion via acting as a molecular sponge to miR-718

**DOI:** 10.1042/BSR-2018-2018_RET

**Published:** 2023-02-27

**Keywords:** breast cancer, lncRNA FBXL19-AS1, miR-718

This Retraction follows an Expression of Concern relating to this article previously published by Portland Press. This article is being retracted from *Bioscience Reports* at the request of the Editor-in-Chief and the Editorial Board following receipt of a notification from a reader, alerting the Editorial Board to images that seem to appear in another paper by Jiang et al. 2018 (https://doi.org/10.1186/s13046-022-02418-x). Specifically, the Figure 4F panels (Ki-67/sh-NC panel vertically flipped) appear as part of the Figure 3C Ki67 sh-Vector and sh-TPT1-AS1 panels of Jiang et al., and the Figure 2F western blots appear the same as the Figure 5H blots of Jiang et al. The authors have been contacted with regards to the retraction and have not responded to the Journal's queries or the concerns raised. Given the extent of the issues raised, the Editorial Board stand by the decision to retract the article.

